# Ecological Momentary Assessment and Intervention Principles for the Study of Awake Bruxism Behaviors, Part 2: Development of a Smartphone Application for a Multicenter Investigation and Chronological Translation for the Polish Version

**DOI:** 10.3389/fneur.2019.00170

**Published:** 2019-03-05

**Authors:** Magdalena A. Osiewicz, Frank Lobbezoo, Alessandro Bracci, Jari Ahlberg, Jolanta Pytko-Polończyk, Daniele Manfredini

**Affiliations:** ^1^Department of Integrated Dentistry, Dental Institute, Faculty of Medicine, Jagiellonian University Medical College, Cracow, Poland; ^2^Department of Orofacial Pain and Dysfunction, Academic Centre for Dentistry Amsterdam, University of Amsterdam and Vrije Universiteit Amsterdam, Amsterdam, Netherlands; ^3^School of Dentistry, University of Padua, Padua, Italy; ^4^Department of Oral and Maxillofacial Diseases, University of Helsinki, Helsinki, Finland; ^5^School of Dentistry, University of Siena, Siena, Italy

**Keywords:** awake bruxism, bruxism, translation, ecological momentary assessment, smartphone, diagnosis

## Abstract

**Objectives:** The aim is to describe the process of translating the smartphone application *BruxApp* into Polish within the context of an ongoing multicenter project on awake bruxism (AB) epidemiology.

**Material and Methods:** An ongoing cooperation involving 11 universities is based on the adoption of the smartphone-based EMA protocol to collect real time report of AB behaviors in the natural environment. The English version of *BruxApp* is adopted as a template for the multi-language translation, according to a step-by-step procedure led by mother-tongue experts in the field. A dedicated web platform for translation (viz., *POEditor*) is used. The process of translation into Polish is here described as an example.

**Results:** There are two software versions available, viz., *BruxApp* and *BruxApp Research*. For both versions, back translation from Polish to English was performed to verify the accuracy of the translation procedure. The validity of the translation has been confirmed by the perfect agreement between the original and back-translated English versions, and the Polish version of *BruxApp* can thus be introduced in the clinical and research setting to get deeper into the study of AB epidemiology in Poland.

**Conclusions:** As far as clinical studies are concerned, the described strategy to record data can be very useful—patients can acknowledge their habits, monitor changes over time, and implement remedial measures. In the field of research, *BruxApp* makes it possible to collect and store a huge amount of data about the epidemiology of different forms of awake bruxism, both at the individual level and at the population level.

## Introduction

Awake bruxism (AB) is a masticatory muscle activity during wakefulness that is characterized by repetitive or sustained tooth contact and/or by bracing or thrusting of the mandible and is not a movement disorder in otherwise healthy individuals (1). The expert consensus panel that provided the above definition also suggested a diagnostic grading to assess both sleep and awake bruxism as “possible,” “probable,” and “definite.” In addition, the panel recognized that most research so far has focused on sleep bruxism (SB), so that knowledge on AB is fragmental due the incomplete description of its epidemiology.

The recent expert consensus paper suggested that a definite diagnosis of AB should be based on electromyographic (EMG) recordings or, as an alternative due to the compliance limitations to perform hour-long EMG recordings of jaw muscles' activity during wakefulness, could rely on Ecological Momentary Assessment (EMA). EMA is a simple method to collect data on patients' self-reported AB. Its introduction in the research setting could allow getting a better understanding of the condition's epidemiology as well as of the condition's relationship with various forms of bruxism, masticatory muscle pain, and temporomandibular joint (TMJ) pain (2). Comparison of data across studies would be implemented by the adoption of a standardized EMA approach and, as discussed in the previous paper, taking advantage of smartphone technology seems to be a rational approach.

Based on the above, this manuscript describes in more detail a software application that is now available to report AB behaviors close in time with the experience (*BruxApp*, BruxApp Team, Pontedera, Italy) (3). As a secondary aim, the paper also introduces an ongoing multicenter cooperation involving, for now, 11 universities on the adoption of a smartphone-based EMA protocol to collect real time report of AB behaviors in the natural environment. For this purpose, the application has been formally translated into more than 20 languages, and a standardized procedure for translations (i.e., using the so-called forward and back translation procedure via web platforms) has been adopted. So far, no formal Polish translation was performed. Hence, the final aim of this article is to present the Polish version of the application *BruxApp*, and to describe the process of its official translation from English into Polish and all other languages.

### BruxApp

The BruxApp introduced the EMA principles in the field of AB by the use of smartphone technology. The idea of EMA was formulated in the eighties as a strategy to overcome the limitations of the traditional quantitative methods used in various psychological studies (4). The common principle of many EMA techniques is that the patient reports in real time the outcome variable under investigation, as in the case of AB behaviors. Such reports can be obtained repeatedly for prolonged periods of observation (5), and the use of a smartphone app fits perfectly with the need for maximizing compliance and simplicity (6). Moreover, smartphone-based EMA approaches could also be implemented in an interventional strategy (i.e., EMI) to educate patients about a behavior that may be negative for their health.

Within these premises, the *BruxApp* application aims to re-educate the patient by reminding him/her to relax his/her muscles and to avoid teeth contact. The application is based on a very simple principle of data recording. Thanks to the sound emitted by the app, the patient is alerted to focus his/her attention on jaw muscles and teeth position at random times during the day to allow a real time report. This enables monitoring the patient's oral behaviors in his/her natural environment.

**Figure 1:**
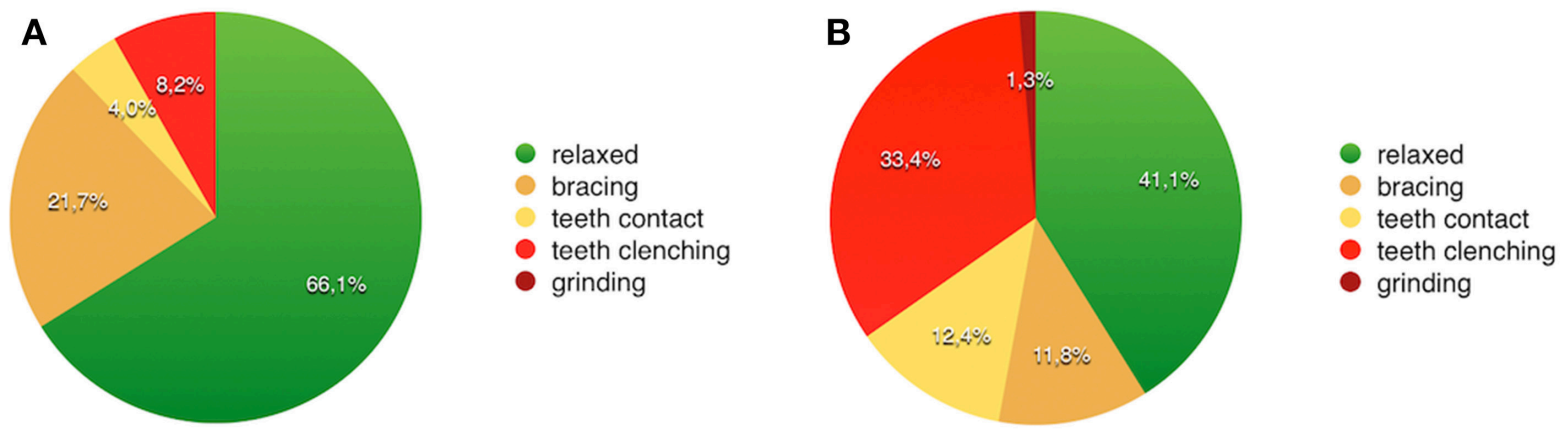
Examples of screenshots of individual AB activities recorded using the BruxApp over 1 week. In the example patient **(A)**, muscles were relaxed in 66.1% of the recorded time, while in in the patient **(B)**, the frequency of relaxed answers was 41.1%. The most frequent AB behaviors are teeth clenching (33.4%) and teeth contact (12.4%).

**Figure 2:**
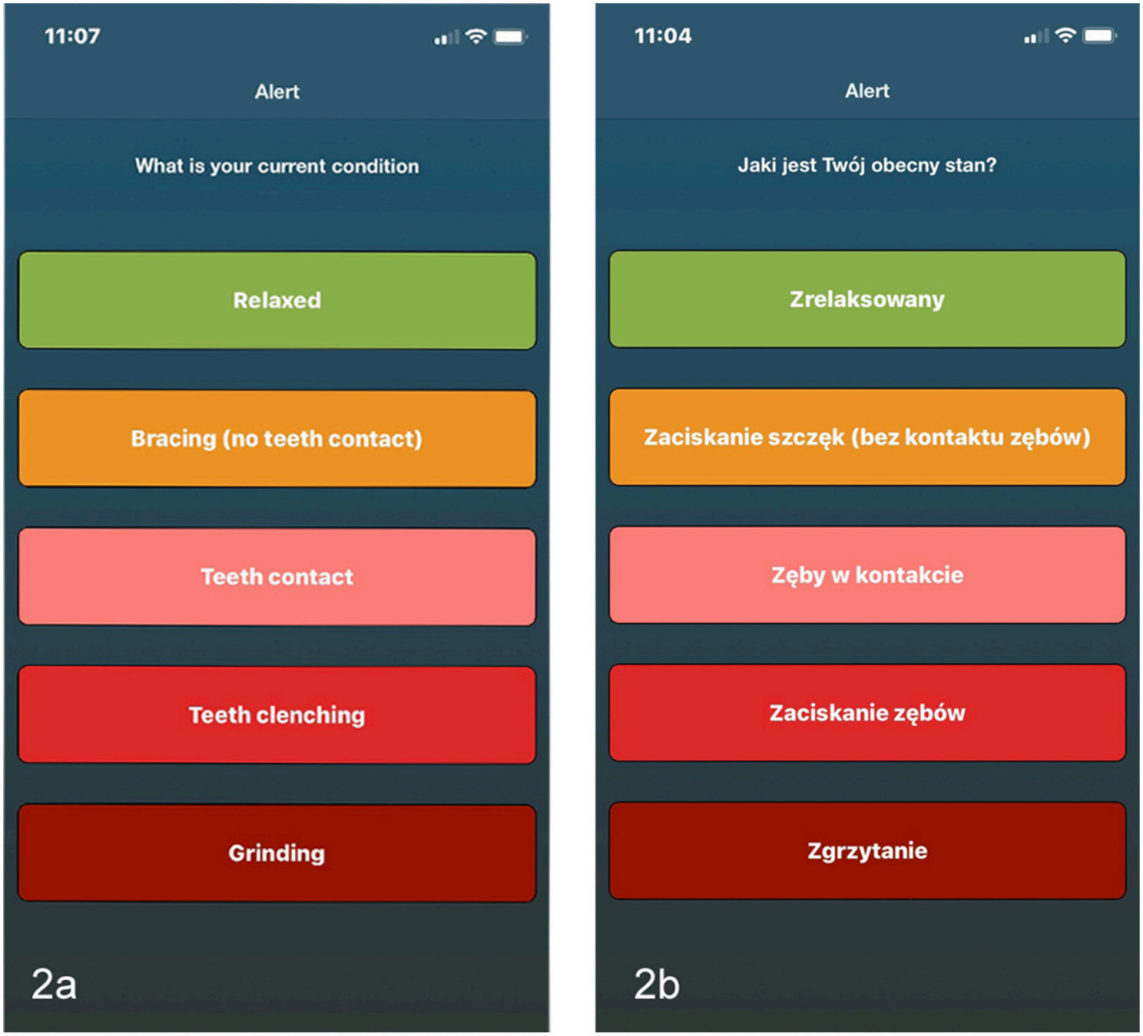
Screenshots of the *BruxApp* application. Comparison of the original English version **(a)** and the Polish one **(b)**, as an example of the translational procedures.

**Figure 3:**
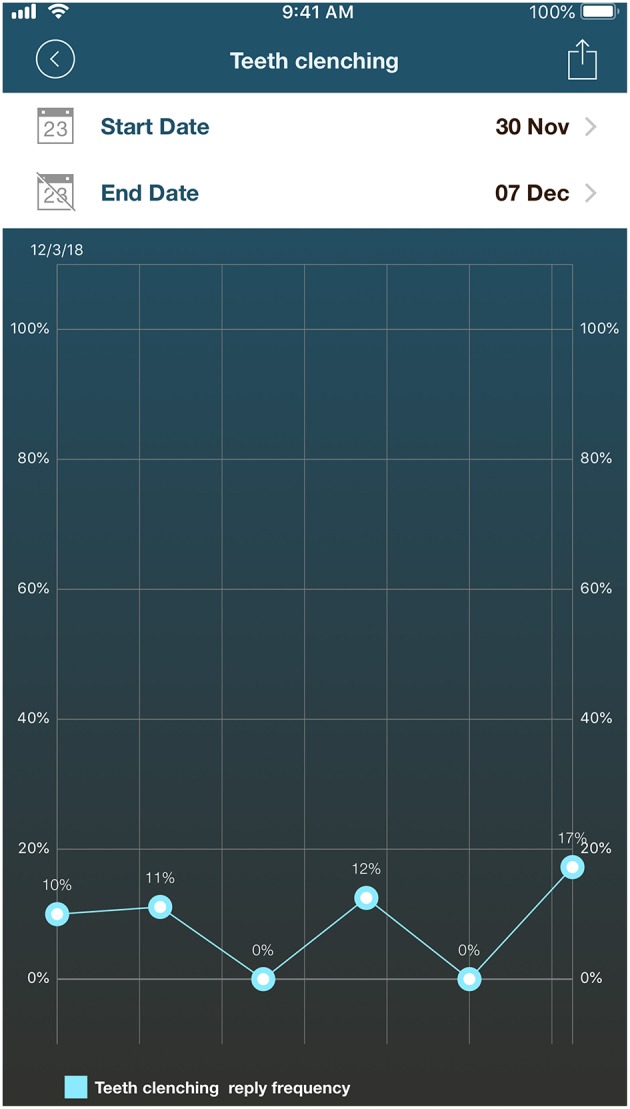
Screenshot of Individual results for the behavior “teeth clenching” recorded using the English version of *BruxApp* for 1 week.

**Figure 4:**
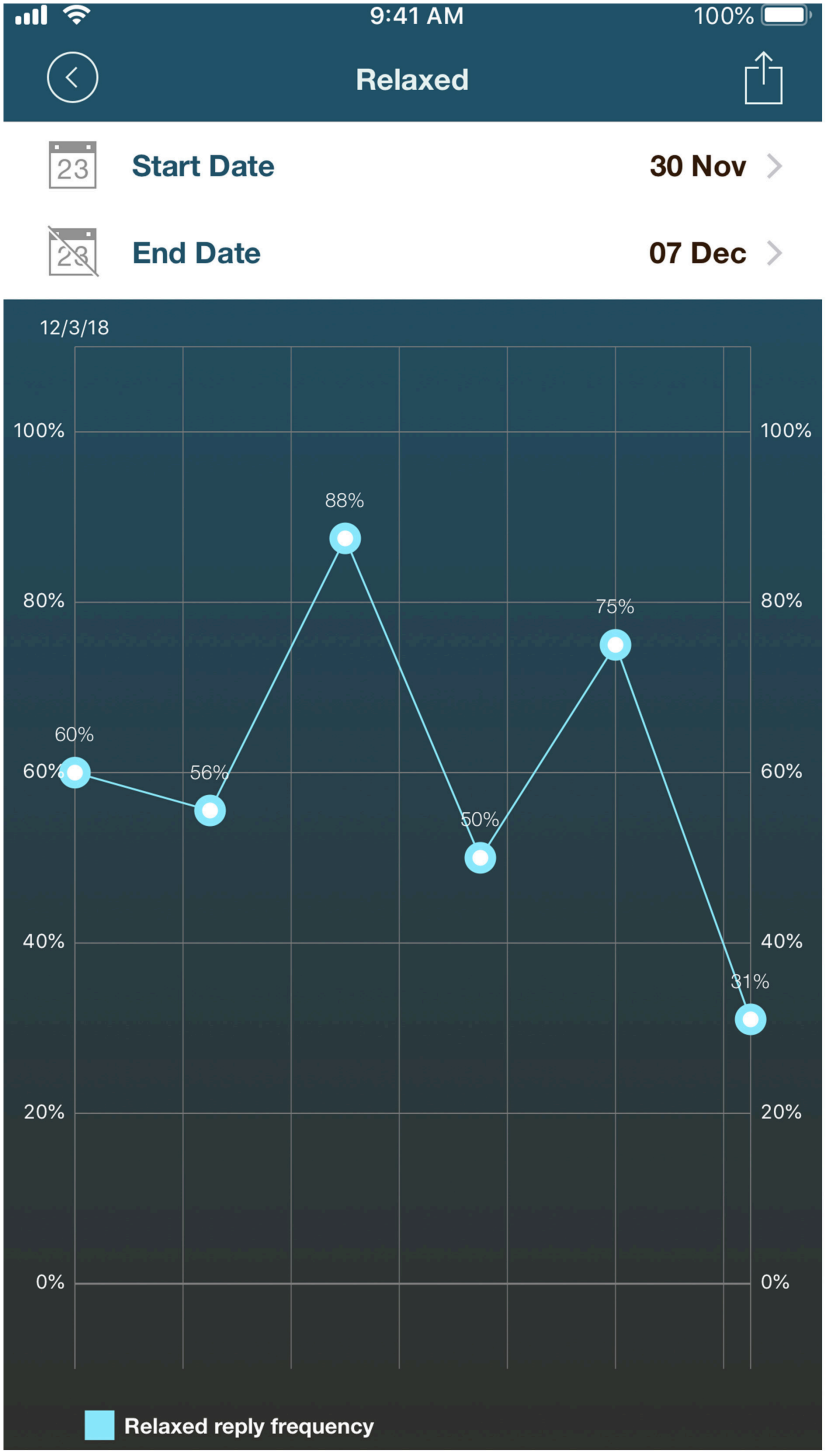
Screenshot of individual results for the “relaxed” answer recorded using the English version of *BruxApp* for 1 week.

When the patient receives the alert, he/she has to identify the current condition from among five options: relaxed jaw muscles, mandible bracing, teeth contact, teeth clenching, or teeth grinding. An additional answer to an item on the presence of facial pain is also required. After that, the patient has to tap on the display and give a real time feedback (Figures 1–4). The application has a very simple and intuitive interface, which can be personalized according to the individual needs and expectations. The default mode provides a 7-day monitoring, which can be extended or/and repeated to collect data for longer periods. Alert sounds are generated at a random frequency, with no predefined time intervals. There are several options designed to allow for the individualization of the alarm setting, such as the number of observation days, the start and end time of the report during the day, and the number of alarms per day.

Importantly, the application also includes an information section on bruxism and an explanation of the objectives of *BruxApp*. As a result, pain in the jaw muscles and/or the temporomandibular joint could be managed within a cognitive-behavioral framework.

### Multicenter Research

The potential number of *BruxApp* users is impressive. According to the data reviewed in 2013 by Manfredini et al. sleep bruxism affects 12% of the general population, whereas awake bruxism occurs in approximately 22–30% of people (7). The latest estimates show that in economically well-developed regions, approximately 35% of people have mobile phones with an application download feature (iOS and Android). There are no exact data on the number of downloads for medical applications, but if one assumes that approximately 10% of patients who are affected by bruxism and who own a smartphone would be willing to download *BruxApp* on their phones, the potential of the application for epidemiological research will be enormous at a worldwide level.

As a result of this impressive potential, an ongoing multicenter cooperation involving 11 universities is currently performed with the adoption of a specific smartphone-based EMA protocol (*BruxApp Research*). Experts and leaders on the topic are coordinating data collection in various languages. *BruxApp* is currently available in the Bosnian, Brazilian, Chinese, Croatian, Dutch, English, Finnish, French, German, Greek, Hebrew, Italian, Japanese, Lithuanian, Polish, Portuguese, and Spanish language. About ten other translations are currently performed, based on the procedure described below.

Training sessions for the leading researchers are organized under the supervision of the main coordinator, who is an expert in primary research and clinical management of bruxism (D.M.). In short, the critical issue is to maximize internal validity of findings, and careful instructions to the patients have to be conveyed as for the recognition of the following five conditions:

- Relaxed jaw muscles: condition of perceived relaxation of jaw muscles, with the jaws kept apart;
- Teeth contact: condition of slight teeth contact like the teeth contact that the subject perceives when a 40 μ articulating paper is put between the dental arches and he/she is asked to slightly keep the teeth in contact to retain it on site. In short, this condition is defined as light touching of teeth when the mouth is closed;
- Teeth clenching: all conditions in which teeth contacts are more marked than the above, and jaw muscles are kept tense;
- Teeth grinding: condition in which the opposite teeth are gnashed or ground, independently of the intensity and direction of antagonist teeth contacts;
- Jaw clenching (without teeth contact): condition of jaw-muscle stiffness or tension like teeth clenching, but with teeth kept apart (i.e., mandible bracing).

The standardization of this message is fundamental to overcome the shortcomings due to the self-reported nature of the gathered data and to implement the comparability of data across different investigations. The ultimate goal of the large-scale multicenter cooperation is to enhance research on AB with the creation of international databases for data storage and consultation. A multi-language platform for multicenter research coordination and implementation of cross-cultural and ethnic comparisons has thus been created, and the translational process is described below.

### Translation Into **Polish**

The English version of *BruxApp* is adopted as a template for the multi-language translation, according to a step-by-step procedure led by mother-tongue experts in the field. As a first step, the Polish bruxism expert (M.O.) was trained by the supervisors of the smartphone—EMA project adopting the software *BruxApp (AB; DM)*. Then, the application was tested by the same expert for the next 2 weeks, before translating the files into Polish. The files describe the objectives and functioning of the application, and contain the *BruxApp* manual. The next step was to translate *strings*, which means sequences of alphanumeric texts in computer programming, which are extremely important for the functioning of the application. All translation procedures were done on a special platform (viz., *POEditor)*, on which English is the reference language. Before the translation was done, the translator had to get acquainted with the *POEditor* manual.

As part of the cultural adaptation process, two bilingual Polish mother-tongue translators translated the original text independently. One translator was a professional in the bruxism field, while the other translator was naïve to this field. In general, when creating an instrument, the selection of two translators who have different knowledge helps in reducing bias and enhancing a more general understanding of the item content. The two independent translations were assessed for differences by the Polish project coordinator (MO), who created a single forward translation. The so-derived Polish forward translation, was sent to an independent bilingual back translator whose native language is English. The back translator was naïve to the source text but had some basic knowledge of the bruxism field.

A reviewer who was a contributing author of the original version of *BruxApp* (AB) compared the back translation to the original version. A document describing all discrepancies between the back translation and the source (English) version was created in order to identify areas of concern in the Polish translation. The forward and back translation steps were then repeated for the discrepant items, followed by an independent review. In the next stage, a committee consisting of a Polish language specialist and two laypersons (one of whom did not know the English language, and one of whom was Polish-English bilingual) reviewed the Polish version of *BruxApp*. The task of the committee was to review the translation with regard to four types of equivalences (somatic, idiomatic, experiential, and conceptual) of the document as to validate it cross-culturally.

The validity of the English-to-Polish translation was confirmed by the perfect agreement between the original and back-translated English versions. This means that the Polish version of *BruxApp* can be introduced in the clinical and research setting to get deeper into the study of AB epidemiology in Poland.

## Discussion

The *BruxApp* was conceptualized based on the idea that bruxism may be considered one of civilization's “phenomena of the new millennium.” In the clinical setting, there are some empirical observations that awake bruxism, exerted in the form of keeping the jaw muscles in a prolonged tension or in the form of repeated teeth contact habit, may be associated with fatigue and pain (8). Cognitive behavioral approaches, which help patients understanding their need to maintain relaxed mastication muscles, are likely the best first-step therapeutic solution. In addition, they could be seen as important strategies to strengthen the positive long-term effects of other treatment protocols.

Therefore, along with the usefulness as an assessment tool, the application may educate the patient to understand the possible consequences of bruxism and may serve as a *biofeedback* strategy with a large clinical potential. Repeated alerts at random intervals are potentially useful as an educational strategy to acquire awareness and reverse the awake bruxism behaviors in patients with TMD pain. When the patient receives an alert, he/she must pay attention to the position of the teeth and the condition of the mastication muscles; doing so, the patient can react properly, for example by relaxing the jaw muscles.

Clinical research protocols should be established to assess the effectiveness of this approach, and it is plausible that the clinical outcomes largely depend on the patient's self-discipline to use the application. Notwithstanding the latter, the data collected with the application are useful, both individually and at the general population level. For instance, at the individual level, the immediate return of the information makes it possible to have a fruitful doctor-to-patient relationship. At the population level, investigators of the various research centers can achieve a clearer picture of the prevalence, incidence, and risk factors of AB. Therefore, using officially translated and culturally adapted versions of *BruxApp* can be helpful for a consistent communication among dental professionals. On this purpose, EMA/AB data gathered in otherwise healthy individuals will provide a dataset for a range of average frequency values of AB behaviors as a function of the different populations in different countries. From there, comparative studies can be performed in selected study samples, also taking into account the time-related variability of the specific AB activities.

## Conclusions

*BruxApp* makes it possible to collect data on awake bruxism behaviors for both clinical and research purposes. In the clinical setting, the data can be useful to help patients acquiring awareness of their habits, monitoring changes over time, and implementing appropriate corrective measures. In the research setting, using an officially translated and culturally adapted version of *BruxApp* provides opportunities to collect a large amount of data about the epidemiology of different forms of awake bruxism, both at the individual and at the population level.

## Author Contributions

MO designed the study, performed the study, wrote, and revised the article. DM supervised and critically revised the article. FL, AB, JA, and JP-P critically revised the article.

### Conflict of Interest Statement

The authors declare that the research was conducted in the absence of any commercial or financial relationships that could be construed as a potential conflict of interest.

## References

[1] Lobbezoo F Ahlberg J Raphael KG Wetselaar P Glaros A Kato T. International consensus on the assessment of bruxism: report of a work in progress. J Oral Rehabil. (2018) 45:837–44. 10.1111/joor.12663 29926505 PMC6287494

[2] Bracci A Djukic G Favero L Salmaso L Guarda-Nardini L Manfredini D Frequency of awake bruxism behaviors in the natural environment. A seven-day, multiple-point observation of real time report in healthy young adults. J Oral Rehabil. (2018) 45:423–9. 10.1111/joor.12627 29574964

[3] Bruxism Smart Application for Devices Available online at: www.bruxapp.info

[4] Shiffman S Stone AA Hufford MR. Ecological momentary assessment. Annu Rev Clin Psychol. (2008) 4:1–32. 10.1146/annurev.clinpsy.3.022806.091415 18509902

[5] Moskowitz DS Young SN. Ecological momentary assessment: what it is and why it is a method of the future in clinical psychopharmacology. J Psychiatry Neurosci. (2006) 31:13–20. 16496031 PMC1325062

[6] Runyan JD Steinke EG. Virtues, ecological momentary assessment/intervention and smartphone technology. Front Psychol. (2015) 6:481. 10.3389/fpsyg.2015.00481 25999869 PMC4422021

[7] Manfredini D Winocur E Guarda Nardini L Paesani D Lobbezoo F. Epidemiology of bruxism in adults. A systematic review of literature. J Orofac Pain. (2013) 27:99–110. 10.11607/jop.921 23630682

[8] Manfredini D Lobbezoo F. Relationship between bruxism and temporomandibular disorders: a systematic review of literature from 1998 to 2008. Oral Surg Oral Med Oral Pathol Oral Radiol Endod. (2010) 109:e26–50. 10.1016/j.tripleo.2010.02.013 20451831

